# Use of Rucaparib Plus Temozolomide in Advanced Uterine Leiomyosarcoma: A Case Report From a Tertiary Centre in India

**DOI:** 10.7759/cureus.115193

**Published:** 2026-08-25

**Authors:** Adhip Arora, Sameer Rastogi, Aman Tilak, Shamim Ahmed, Ruchi Rathore

**Affiliations:** 1 Department of Medical Oncology, All India Institute of Medical Sciences, New Delhi, New Delhi, IND; 2 Department of Nuclear Medicine, All India Institute of Medical Sciences, New Delhi, New Delhi, IND; 3 Department of Pathology, All India Institute of Medical Sciences, New Delhi, New Delhi, IND

**Keywords:** parp-inhibitors, rucaprib, temozolomide, uterine leiomyosarcoma, uterine sarcoma

## Abstract

The treatment landscape for metastatic uterine leiomyosarcoma has expanded in recent years with the introduction of newer chemotherapeutic and targeted therapies; however, effective treatment options remain limited. We present the case of a woman in her early 60s with metastatic uterine leiomyosarcoma who had received five lines of therapy for metastatic disease. She was subsequently treated with rucaparib 400 mg twice daily on days one through seven of a 28-day cycle and temozolomide 120 mg once daily on days one through seven of the same cycle, with subsequent dose reduction of both agents because of hematological toxicity. After three months of treatment, she achieved a complete metabolic response on fluorodeoxyglucose-positron emission tomography/computed tomography (FDG-PET/CT). After eight months of treatment, she developed progressive disease. Next-generation sequencing for the homologous recombination repair deficiency panel was attempted but was unsuccessful because of degraded DNA; therefore, her homologous recombination deficiency status could not be confirmed. This case describes clinical activity with rucaparib plus temozolomide in a heavily pretreated patient with advanced uterine leiomyosarcoma and highlights the need for further investigation of this therapeutic approach.

## Introduction

Uterine leiomyosarcoma (uLMS) is a rare and aggressive uterine malignancy and represents the most common histologic subtype of uterine sarcoma [1]. Although it constitutes only about 1% of all uterine cancers, it is responsible for a significant proportion of deaths related to uterine malignancies, primarily due to its tendency to metastasize early and its limited response to standard treatments [2]. In contrast to endometrial carcinoma, which is often detected early owing to symptoms like abnormal bleeding, uLMS is usually diagnosed at a more advanced stage. The exact mechanisms underlying the development of uLMS remain unclear. However, molecular research has revealed frequent genetic alterations - particularly mutations in TP53, RB1, and ATRX - along with significant chromosomal instability. These findings are believed to play a role in the tumour’s aggressive clinical course [3].

uLMS typically spreads to the lungs, liver, abdomen, and pelvis [4]. Doxorubicin with trabectedin is the first-line therapy for advanced leiomyosarcoma (median overall survival (OS) of 33 months vs 24 months in the doxorubicin alone arm) [5]. In unfit patients, single-agent doxorubicin can be considered as the first-line treatment. Gemcitabine with docetaxel is usually the second-line treatment in these cases; however, it may be given upfront in a selected group of patients. It offers a median progression-free survival (PFS) of 6.2 months and a median OS of 17.9 months [6]. Trabectedin and pazopanib are approved for later lines of therapy.

uLMS cases have more somatic BRCA2 mutations (up to 10% of cases) as compared to non-uterine LMS [7]. Mutations in the homologous recombination (HR) DNA repair pathway have been observed in approximately 10% of uLMS cases [8].

HR encompasses a network of interconnected pathways responsible for repairing DNA breaks and interstrand crosslinks. In uLMS, somatic homozygous deletions of BRCA2 are commonly observed, resulting in HR deficiency. PARP inhibitors function by competing with NAD+ at the catalytic domain of PARP, thereby inhibiting its enzymatic activity and preventing the formation of PAR polymers [9]. This disruption impairs DNA replication, repair, chromatin remodelling, and gene transcription in cells with BRCA1/2 mutations, ultimately leading to cell death [10].

Olaparib targets tumors with defective HR by inhibiting DNA repair, leading to cell death in BRCA-mutated cancers [10]. In uLMS, where BRCA2 loss occurs in a subset of patients, this approach may offer therapeutic benefit. A series of four such patients treated with olaparib showed encouraging results - three had prolonged disease stability, and one demonstrated partial tumor shrinkage, despite prior treatment failures [7].

Olaparib with temozolomide has demonstrated a clinically meaningful benefit post first-line therapy, more in HR-deficient tumours [11]. In a phase II, single-arm study by Ingham et al., 22 patients with advanced uLMS received olaparib 200 mg twice daily on days one through seven of a 21-day cycle and temozolomide 75 mg/m² once daily on days one through seven. The objective response rate was 27%, and the median PFS was 6.9 months. Exploratory analysis showed a longer median PFS in patients with HR-deficient tumors compared with HR-proficient tumors (11.2 vs 5.4 months) [11]. Although disease-specific clinical evidence is currently available predominantly for olaparib in combination with temozolomide, rucaparib is another PARP inhibitor with clinical experience in combination with cytotoxic chemotherapy, including temozolomide [12]. However, evidence supporting rucaparib in uLMS remains limited.

## Case presentation

We present a case report of a woman in her early 60s, whose baseline presentation was two years ago. She developed left-sided abdominal pain, associated with nausea and vomiting, for three months. For these complaints, she visited a general practitioner and was advised to take some antacids. She had persistent symptoms, for which she was further evaluated and found to have a uterine mass with lung nodules and mediastinal nodes.

**Figure 1:**
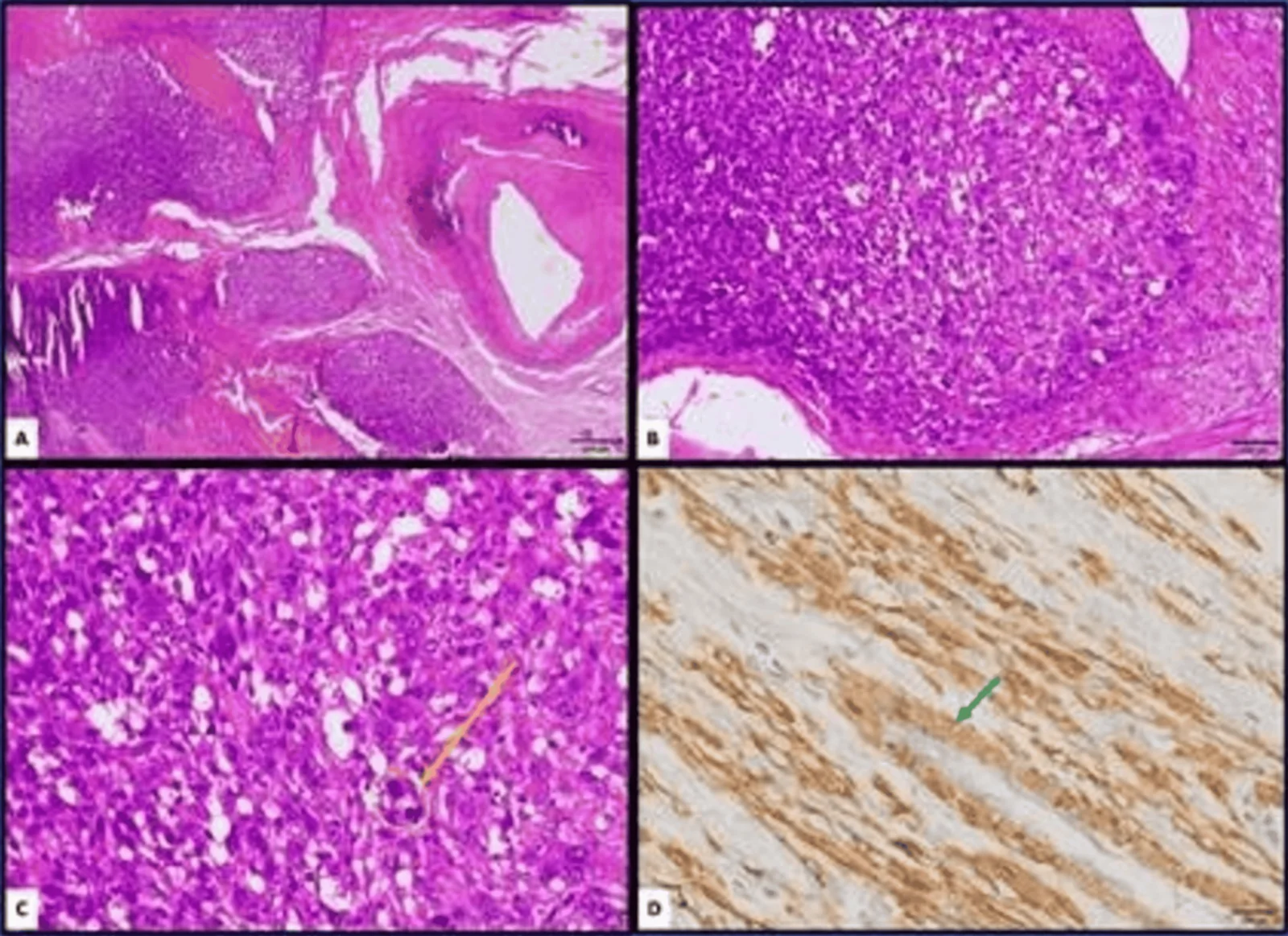
Histopathological and immunohistochemical features of lung metastasis Histopathological examination of the lung metastasis. (A) Low-power photomicrograph showing metastatic tumor nodules composed of spindle-shaped cells with surrounding areas of hemorrhage (hematoxylin and eosin [H&E], ×40). (B, C) Higher-power views demonstrating pleomorphic spindle cells with hyperchromatic, pleomorphic nuclei, coarse clumped chromatin, and brisk mitotic activity (arrow) (H&E, ×200 and ×400, respectively). (D) Immunohistochemistry showing diffuse cytoplasmic positivity for smooth muscle actin (SMA) in the tumor cells, with perivascular smooth muscle serving as an internal positive control (arrow) (SMA immunohistochemistry, ×200).

She underwent biopsy for the lung mass. Histopathological examination is shown in Figure 1.

She underwent endobronchial ultrasound (EBUS)-guided fine-needle aspiration cytology (FNAC), which was positive for Mycobacterium tuberculosis. She was started on four-drug antitubercular therapy (ATT).

She underwent exploratory laparotomy, and post-operative histopathology was suggestive of conventional (spindle cell) uLMS, infiltrating the left paracervical tissue. On immunohistochemistry testing, the tumor cells were positive for SMA and SMMH while negative for CK, CD10, CYCLIN D1, and CD34. A diagnosis of metastatic uLMS was made. Based on this histopathology report she was started on injection gemcitabine and docetaxel chemotherapy alongside ATT.

She was now referred to our centre and we worked up the case. Positron emission tomography/computed tomography (PET/CT) was done and a biopsy was reviewed by our sarcoma pathologist.

She received her first cycle of gemcitabine (675 mg/m2; D1 & D8) and docetaxel (75 mg/m2 D1 only) of a 21-day cycle on 18/05/2022. After receiving three cycles of gemcitabine and docetaxel, a repeat PET scan was done, which was suggestive of progressive disease. She was started on second-line therapy, i.e., doxorubicin (75 mg/m2 on day 1) with dacarbazine (400 mg/m2 D1-D3) in a 21-day cycle.

For response assessment, a fluorodeoxyglucose (FDG) PET scan was done after six cycles of chemotherapy, which was suggestive of partial response. She was kept under observation after that. After a treatment-free interval of three months, her disease progressed. She was given third-line therapy of tablet pazopanib 600 mg once a day.

She was given an aromatase inhibitor (letrozole 2.5 mg once a day) with pazopanib. She did well for the next four months, following which a reassessment FDG PET/CT scan was done, which was suggestive of progressive disease. She was started on trabectedin (1.4 mg/m2 infusion over 24 hours) as the fourth-line treatment. She received a total of nine cycles, following which she again progressed, giving her a progression-free duration of seven months. PET scan was done after seven months, which was suggestive of disease progression. She was then given eribulin at a dose of 1.4 mg/m2 D1, D8 of a 21-day cycle. After three cycles, her disease again progressed, giving a progression-free interval of only three months.

Next-generation sequencing (NGS) for the homologous recombination repair (HRR) panel was attempted but failed in view of degraded DNA. Therefore, the patient's homologous recombination deficiency (HRD) status could not be confirmed.

We planned to give her olaparib and temozolomide, but in view of financial constraints we gave her rucaparib at a dose of 400 mg twice a day on days one to seven of a 28-day cycle. The seven-day intermittent rucaparib schedule was extrapolated from prior clinical experience with intermittent oral rucaparib administration in combination with cytotoxic therapy [13].

Doses of rucaparib and temozolomide had to be reduced to 200 mg twice a day and 120 mg once a day, respectively, on day one to day seven of a 28-day cycle in view of persistent haematological toxicity in the form of grade 3 anaemia and thrombocytopenia and grade 3 fatigue.

**Figure 2:**
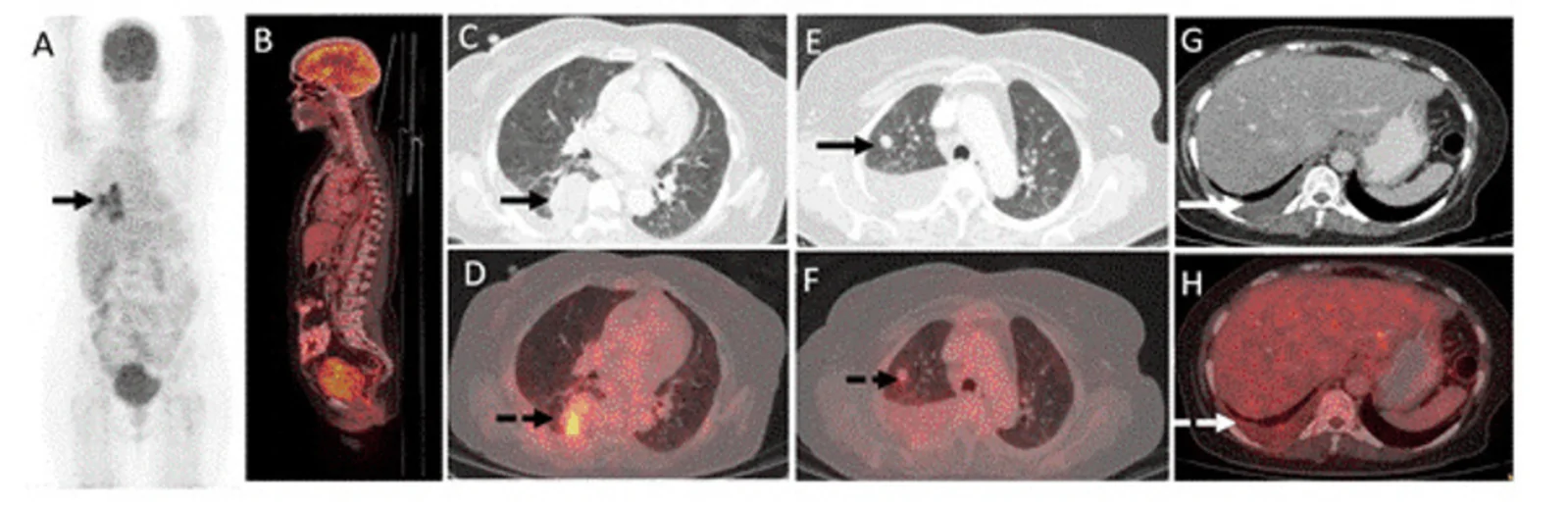
Pre-treatment FDG PET/CT demonstrating metastatic pulmonary and pleural disease Pre-treatment ^18F-FDG PET/CT images. (A) Maximum intensity projection (MIP) image demonstrating increased FDG uptake in the right hemithorax (black arrow). (B) Sagittal fused PET/CT image showing post-hysterectomy status without evidence of FDG-avid local residual or recurrent disease. (C, E) Axial CT images (black arrows) and (D, F) corresponding fused PET/CT images (black dotted arrows) demonstrating FDG-avid metastatic right lung nodules. (G) Axial CT image (white arrow) and (H) corresponding fused PET/CT image (white dotted arrow) showing FDG-avid right pleural effusion. FDG: fluorodeoxyglucose, PET/CT: positron emission tomography/computed tomography

**Figure 3:**
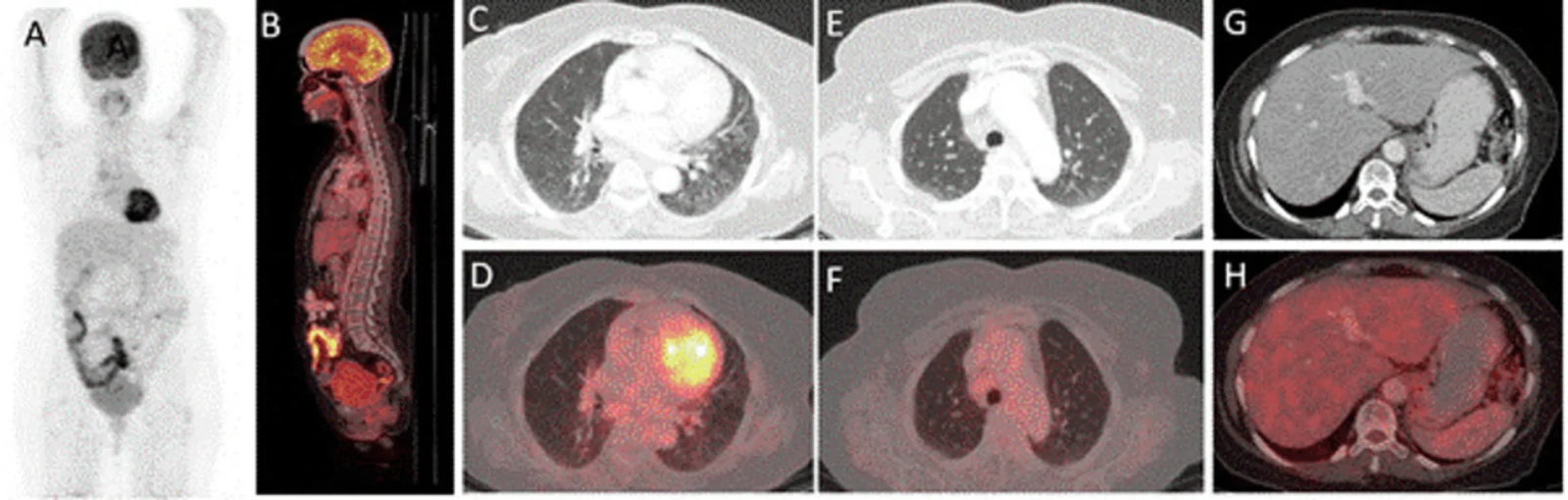
Post-treatment ^18F-FDG PET/CT demonstrating complete metabolic response after temozolomide and rucaparib Post-treatment ^18F-FDG PET/CT obtained after three cycles of temozolomide and rucaparib. (A) Maximum intensity projection (MIP) image showing complete resolution of the previously noted foci of increased FDG uptake in the right hemithorax. (B) Sagittal fused PET/CT image demonstrating post-hysterectomy status without evidence of FDG-avid local residual or recurrent disease. (C, E) Axial CT images and (D, F) corresponding fused PET/CT images showing complete resolution of the previously identified FDG-avid metastatic right lung nodules. (G) Axial CT image and (H) corresponding fused PET/CT image demonstrating complete resolution of the previously noted right pleural effusion. Overall findings are consistent with a complete metabolic response. FDG: fluorodeoxyglucose, PET/CT: positron emission tomography/computed tomography

After three cycles, response assessment was done and was suggestive of a complete metabolic response. PET scans before and after three cycles of temozolomide and rucaparib are shown in Figures 2, 3, respectively.

She received eight cycles and a PET/CT scan was done, following which she showed disease progression. Rucaparib with temozolomide gave her a progression-free interval of eight months. Table 1 shows the timeline and clinical response across various lines of therapy.

**Table 1:** Treatment Timeline and Clinical Response Across Six Lines of Systemic Therapy Treatment timeline and clinical response across successive lines of systemic therapy. The figure depicts the sequence of six lines of systemic treatment, treatment duration, best response, and progression-free interval. Following progression on five prior systemic regimens, treatment with rucaparib plus temozolomide in the sixth line resulted in a complete metabolic response, which was maintained for eight months. Dose reduction of rucaparib from 400 mg twice daily to 200 mg twice daily and temozolomide to 120 mg once daily was subsequently undertaken.

Line	Treatment	Treatment duration	Best response	Progression-free interval
1st line	Gemcitabine 675 mg/m² D1 & D8 + docetaxel 75 mg/m² D1, every 21 days	3 cycles	Progressive disease	-
2nd line	Doxorubicin 75 mg/m² D1 + dacarbazine 400 mg/m² D1-D3, every 21 days	6 cycles	Partial response	3-month treatment-free interval
3rd line	Pazopanib 600 mg once daily + letrozole 2.5 mg once daily	4 months	Progressive disease	4 months
4th line	Trabectedin 1.4 mg/m² infusion over 24 hours	9 cycles	Progressive disease	7 months
5th line	Eribulin 1.4 mg/m² D1 & D8, every 21 days	3 cycles	Progressive disease	3 months
6th line	Rucaparib 400 mg twice daily D1-7 + temozolomide; subsequent dose reduction to rucaparib 200 mg twice daily and temozolomide 120 mg once daily D1-7, every 28 days	8 cycles	Complete metabolic response	8 months

Outcome and follow-up

She received eight cycles of rucaparib and temozolomide, following which FDG PET/CT showed progressive disease, giving a progression-free interval of eight months. She was subsequently started on regorafenib. At the latest follow-up, she had received regorafenib for approximately two months and remained clinically stable without any new symptoms. A repeat FDG PET/CT was planned every three months, with continuation of regorafenib until disease progression. Objective response assessment on regorafenib had not yet been performed at the time of reporting.

## Discussion

uLMS is an uncommon and aggressive tumor, accounting for approximately 1% of all soft tissue malignancies of the uterus. It is known for its aggressive nature, high chances of recurrence, and metastasis. The prognosis is generally poor, with a five-year survival rate ranging from 10 to 15% when initially diagnosed with metastatic disease [1].

In a phase 2 single-arm, open-label, multicentric study, 22 patients with advanced uLMS who had progressed on first-line therapy were given olaparib (200 mg orally twice a day on days one through seven in a 21-day cycle) and temozolomide (75 mg/m2 orally once daily days one through seven). The overall response rates were 27% (six of 22), with a median PFS of 6.9 months (95% CI, 5.4 months to not estimable). Median PFS was 11.2 months versus 5.4 months (p=0.05) in HR deficient versus HR proficient. Overall response rate was 27%, best objective response being a partial response. None of the patients achieved a complete response. Haematological toxicity was the most common adverse event. Eight patients required dose reduction in olaparib, and two required dose reduction in temozolomide [11].

Although disease-specific clinical evidence is currently available predominantly for olaparib in combination with temozolomide, rucaparib is another PARP inhibitor with clinical experience in combination with cytotoxic chemotherapy, including temozolomide [12]. Intermittent oral rucaparib at a dose of 400 mg twice daily on days one through seven has also been evaluated in combination with cytotoxic therapy [13].

Our patient had relapsed after multiple lines of therapy. NGS for the HRR panel was tried but failed due to degraded DNA, and therefore her HRD status could not be confirmed. We gave her rucaparib instead of olaparib with temozolomide. She did develop haematological toxicities, which required dose reduction.

In an exploratory analysis, patients with HRD tumors experienced a longer median PFS of 11.2 months, compared to 5.4 months in those with HR-proficient tumors (P = .05) [11].

Our patient was also previously heavily treated and had progressed after five lines of therapy. NGS for the HRD panel was tried but failed due to degraded DNA. She was started on rucaparib and temozolomide; doses were reduced in view of haematological toxicities. She achieved a complete metabolic response after initiation of inhibitors with an alkylating agent (temozolomide). She had a progression-free interval of about eight months, which is quite similar to that reported by Matthew Ingham et al. [11].

Olaparib shows reduced effectiveness in HRD-proficient tumors because they do not have the DNA repair weakness targeted by PARP inhibitors. The combination of olaparib with temozolomide showed some efficacy even in non-HRD tumors, although the response was more robust in HRD-positive cases. This combination might extend the benefit of olaparib beyond BRCA- or HRD-positive tumors [11].

Although olaparib is most effective in BRCA-mutant or HRD-positive tumors, it may still offer some benefit in certain non-BRCA tumors, especially when used in combination with agents such as temozolomide [11].

To our knowledge, there is no data from the Indian subcontinent on the use of PARP inhibitors with temozolomide in advanced uLMS. This case report provides encouragement on use of these agents in those who progress on the first line and are either BRCA mutant or have HRD.

## Conclusions

This case highlights the potential role of combining a PARP inhibitor with temozolomide in a heavily pretreated patient with advanced uMLS. Treatment with rucaparib and temozolomide resulted in a complete metabolic response and a progression-free interval of eight months despite progression after multiple previous lines of therapy. However, HRD status could not be confirmed because of failure of NGS due to degraded DNA. Therefore, the response cannot be attributed specifically to HRD. Further studies are required to establish the efficacy, optimal dosing schedule, and patient selection for PARP inhibitor-temozolomide combinations in advanced uLMS.
